# Supplementary material for: Sex as a moderator of the relationship between hip abduction strength and muscle activation during single-leg stance
Source: PLoS One. 2025 Sep 8;20(9):e0331553. doi: 10.1371/journal.pone.0331553 (PMC12416692; doi:10.1371/journal.pone.0331553)
Supplement: S1 Data — (PDF) [file pone.0331553.s001.pdf]

age	sex	body mass	height	BMI	RM_abduction_absolute
32	2	80.1	1.69	28.05	10
33	1	83.6	1.75	27.3	11.3
28	1	85.7	1.71	29.31	17
30	1	91	1.77	29.05	16
27	1	77.4	1.77	24.71	18
33	2	70.4	1.74	23.25	15
29	1	89.6	1.79	27.96	23
22	1	82.2	1.81	25.09	18
20	1	92.4	1.74	30.52	20
26	1	86.5	1.85	25.27	19
31	2	60	1.61	23.15	16
18	2	67.4	1.77	21.51	13.5
33	1	66	1.67	23.67	16.2
27	1	69.7	1.75	22.76	16.7
26	1	84.1	1.74	27.78	17
27	1	103.4	1.78	32.63	24
22	1	77.7	1.71	26.57	20
25	2	53.4	1.55	22.23	14
27	2	61.2	1.76	19.76	15
30	1	101.5	1.83	30.31	22
23	1	102.2	1.81	31.2	23
24	2	70.3	1.57	28.52	15
30	1	84.1	1.81	25.67	18
34	2	52.8	1.52	22.85	11
24	2	54.9	1.53	23.45	12
19	2	54.4	1.53	23.24	12
24	2	55.7	1.59	22.03	12
25	2	64.7	1.71	22.13	13
22	1	86.2	1.81	26.31	21
26	1	80.7	1.77	25.76	21
32	2	46.6	1.5	20.71	11
31	2	65.5	1.66	23.77	7
23	2	60.2	1.6	23.52	13
22	2	60.1	1.65	22.08	13
24	2	51.4	1.56	21.12	10
23	2	52.8	1.75	17.24	10

1 - Male
2 - Female

normalizedRM_abduction (body mass)	Gmed_amplitude (%MVC)	TFL_amplitude (%MVC)
0.12	27.06	2.73
0.14	7.17	1.16
0.2	6.98	2.03
0.18	8.91	2.47
0.23	10	2.36
0.21	7.4	7.06
0.26	9.41	4.18
0.22	14.04	3.31
0.22	8.48	39.9
0.22	13.75	4.87
0.27	2.63	6.96
0.2	4.66	23.54
0.25	3.78	7
0.24	7.84	0.82
0.2	7.93	12.37
0.23	1.56	14.61
0.26	5.02	2.22
0.26	5.9	3.18
0.25	9	1.42
0.22	2.86	1.49
0.23	4.62	0.51
0.21	5.95	2.66
0.21	5.47	0.65
0.21	7.83	3.3
0.22	8.38	1.8
0.22	8	19.31
0.22	10.26	4.2
0.2	7.54	4.4
0.24	5.6	0.35
0.26	2.03	0.95
0.24	17.79	5.81
0.11	26.52	4.89
0.22	6.26	1.36
0.22	9.27	6.72
0.19	13.08	4.47
0.19	6.72	2.52
