# Supplementary material for: Sex as a moderator of the relationship between hip abduction strength and muscle activation during single-leg stance
Source: PLoS One. 2025 Sep 8;20(9):e0331553. doi: 10.1371/journal.pone.0331553 (PMC12416692; doi:10.1371/journal.pone.0331553)
Supplement: Attachment — Submitted filename: Response to reviewer and editor_R1.docx [file pone.0331553.s003.docx]

**LETTER TO EDITOR AND REVIEWERS**

Dear Valentina Graci,

We would like to thank you and the reviewers for the careful and constructive review of our manuscript entitled **SEX AS A MODERATOR OF THE RELATIONSHIP BETWEEN HIP ABDUCTION STRENGTH AND MUSCLE ACTIVATION DURING SINGLE-LEG STANCE**. Please, find the attached revised manuscript containing the suggested modifications (in red) suggested by the reviewer. All comments are addressed below on a point-by-point basis.

Kind regards,

Rodrigo Rodrigues (on behalf of the authors)

REVIEWER #1

**Reviewer:** Dear Authors,

Thank you for the opportunity to review your manuscript. This study investigates an important and timely topic: whether sex moderates the relationship between hip abduction strength and muscle activation during a single-leg stance. The research question is well-motivated, and the finding of a sex-specific negative association in the gluteus medius is intriguing. The manuscript is generally well-written.

However, I have identified several major issues that need to be addressed before the manuscript can be considered for publication

**Author:** Thank you for your comments. We believe that the revised version has been substantially improved.

**Reviewer:** Major Comments

1.The normalization of the TFL EMG data is a significant concern. The authors state that EMG amplitude was normalized to the MVIC of the GMed. As these are two different muscles with distinct functions and activation capacities, normalizing TFL activity to GMed's MVIC is methodologically inappropriate and likely invalidates all results reported for the TFL. The authors should either provide a strong justification for this unorthodox method or, ideally, re-normalize the TFL data using its own MVIC if that data is available. Otherwise, all analyses and conclusions regarding the TFL should be considered for removal from the manuscript.

**Author:** Thank you for your valuable observation. We apologize for the mistake. TFL EMG during MVIC was not clearly mentioned in the original version. All TFL data during single-leg stance were normalized to values of TFL obtained during MVIC of the hip abductors. This has been corrected in the revised manuscript.

**Reviewer:** 2. The regression models do not appear to control for important potential confounders such as body mass and training history, which are critical confounders in strength analyses comparing males and females. I recommend including body size or composition covariates in the model or re-analyzing strength as a relative measure (e.g., normalized to body mass or lean mass) to ensure that the observed effects are not driven by body size alone.

**Author:** Thank you for the excellent suggestion. We conducted the analysis using 1RM normalized by body mass and observed the same result. Therefore, we included both analyses in the manuscript but retained only the graphs with absolute 1RM.

**Reviewer:** 3. The discussion repeatedly frames the increased GMed activation in weaker females as a compensatory strategy for pelvic stabilization. However, this interpretation assumes a functional outcome (i.e., improved pelvic control) that was not directly measured. Without kinematic data or stability-related performance outcomes, such as pelvic drop or trunk sway, this explanation remains speculative. Please consider revising the language to reflect that this is a hypothesis rather than a demonstrated mechanism, or alternatively, support the claim with additional evidence or references.

**Author:** Thank you for your suggestion. We addressed this point in the manuscript by including a reference that supports this mechanism.

**Reviewer:** 4. With only 36 participants and a sex-based interaction model, the power to detect interaction effects may be limited, especially given the potential data variance between groups. This raises concerns about Type II errors for nonsignificant findings (e.g., in males or for TFL) and potential overestimation of the observed effects in females. I suggest including a post-hoc power analysis for the interaction term, or at least discussing the limitations imposed by the sample size more explicitly in the Discussion section.

**Author:** Thank you. We inserted the post-hoc power analysis in the new version.

**Reviewer:** Minor Revisions

1.For transparency and to assess estimate precision, I recommend reporting 95% confidence intervals (CIs) alongside β coefficients and p-values in all regression tables and/or main results text.

**Author:** Thank you. We inserted in the new version.

**Reviewer:** 2. The manuscript does not report how failure to maintain single-leg stance was defined, or whether any trials were excluded due to poor task execution. This information is important for reproducibility. Please add a clear definition of task success/failure and any related exclusion criteria in the Methods section.

**Author:** Thank you. We inserted in the new version.

**Reviewer:** 3. It is unclear whether participants performed the single-leg stance with eyes open or closed, which could affect balance strategy and neuromuscular activation. Please specify the visual condition used during testing and, if not standardized, discuss it as a limitation.

**Author:** Thank you. We clarified in the new version.

**Reviewer:** 4. Although the interaction between sex and strength was not significant for TFL, it would be helpful to report or visualize sex-specific regression slopes, for comparison and transparency. If data permit, I suggest including a supplemental figure or text description.

**Author:** Thank you for your suggestion. We have updated the figure in the revised version to incorporate these changes.

**Reviewer:** 5. The paper does not report whether regression assumptions were tested, such as residual normality, homoscedasticity, or multicollinearity. Please include a brief statement indicating that these assumptions were verified, or report diagnostics if available.

**Author:** Thank you. We clarified in the new version.

**Reviewer:** 6. Since multiple regression models were used to test similar hypotheses for GMed and TFL, a correction for multiple testing (e.g., Bonferroni, Holm) may be warranted. If not applied, please justify this choice and address it as a potential limitation.

**Author:** Thank you for your suggestion. We inserted in the new version.

**Reviewer:** 7. The 1RM estimation from submaximal trials is less common than MVIC in EMG studies and may introduce variability. While the authors cite evidence for its validity, a brief justification for selecting this method over MVIC would strengthen the Methods section.

**Author:** Thank you for your question. Unfortunately, we did not have access to equipment for measuring force during MVIC. Therefore, we relied solely on EMG signal amplitude and used the 1RM test for strength estimation.

**Reviewer:** 8. The authors briefly mention lower tendon stiffness in females as a potential reason for increased GMed activation but do not provide enough detail. Expanding this part of the discussion would enhance the physiological plausibility of the interpretation.

**Author:** Thank you for your suggestion. We have improved this aspect in the revised version.

**Reviewer:** 9. Figure 1 provides a helpful visualization of the data. To further improve its clarity and impact, I suggest the authors add the regression lines for each group (males and females) to both plots. Including the 95% confidence bands for these lines would also be beneficial for interpreting the strength and precision of the observed associations.

**Author:** Thank you for your suggestion. We have updated the figure in the revised version to incorporate these changes.

**Reviewer:** 10. In regression models with significant interaction terms, interpreting main effects independently can be misleading. Please consider rephrasing or de-emphasizing statements about the overall effect of strength across sexes, and focus interpretation within each subgroup.

**Author:** Thank you. In the revised version, when a significant interaction was found, we did not report the main effects.

**Reviewer:** 11. The manuscript does not include a Data Availability Statement. The PLOS ONE policy requires such a statement to clarify for readers how the data can be accessed. Please add a formal statement detailing where the data is located or explaining any restrictions on its availability.

I look forward to seeing a revised version of this manuscript.

**Author: Thank you for mentioning this point. We have added all data related to the manuscript as supplementary material and have added the statement accordingly.**

**“All data are in the manuscript and/or supporting information files.”**

*****************************************************************************

REVIEWER #2

**Reviewer:** I appreciate the opportunity to review the manuscript entitled, “SEX AS A MODERATOR OF THE RELATIONSHIP BETWEEN HIP ABDUCTION STRENGTH AND MUSCLE ACTIVATION DURING SINGLE-LEG STANCE” submitted to PLOS One. This study examined the impact of sex on hip abductor muscle function in the context of single leg stance as it relates to maximal muscle strength and muscle activation. The authors found that lower hip abduction strength was associated with greater activation of the glutes medius muscle in females but not males. The author argues this could represent a sex -specific compensatory strategy for this single leg stance control mechanism. Overall, this is a well written manuscript and in general the data supports the conclusion. I have a few questions and comments I would like the authors to address in terms of the methods and added discussion on the generalizability of the findings to clinical practice. I have provided section by section comments for the authors to consider and address.

Abstract: No edits

Introduction:

Overall, the introduction is well written and organized.

**Author:** Thank you!

**Reviewer:** Methods:

Did the authors conduct an a priori power analysis to determine an adequate sample size for this study? If not, please justify the rationale for not doing one and add this to the manuscript.

**Author:** Thank you for your question. As we did not find similar studies to inform an a priori sample size calculation, we opted for a convenience sample. However, we conducted a post hoc power analysis, which has been included in the Discussion section of the revised manuscript.

**Reviewer:** Lines 150-154 – Although the authors provide a reference with the detailed test description, I would add a short sentence or two that summarizes the methods of this estimated on rep max test. You must consider readers who are clinicians and do not have access to these important references for understanding the context of your study, especially since this method is a cornerstone of this investigation as it is how you measure muscle strength.

**Author:** Thank you for your suggestion. In the new version, we improved the explanation of 1RM test.

**Reviewer:** Lines 158 – I would briefly state that the PROCESS macro is an open-source package used for moderation analysis.

For example, I would just add: “…a moderation analysis was conducted using the PROCESS macro, model 1(version 4.0), which is an open-source package used for moderation analysis.”

**Author:** Thank you for your suggestion. In the new version, we included this statement.

**Reviewer:** Results:

Figure 1 – It would be helpful if the authors added trendlines indicating the relationship for each group, this would include showing the different slopes of the relationship.

Did the authors assess the data in figure 1 for outliers? This was not mentioned in the statistical analysis section. Linear models such as these are highly sensitive to outliers especially small samples.

**Author:** Thank you for the question. We have updated the figure accordingly. Additionally, outlier analysis in the regression was performed using standardized residuals, leverage values, and Cook’s distance. A description of this procedure has been included in the revised version of the manuscript.

**Reviewer:** Discussion:

The discussion does clearly report on the main findings and discusses them in the context of the other literature related to these findings. However, I would like to suggest that the authors consider their finding in the context of single limb control during a dynamic task like walking or single leg squat tasks. Do the authors think the relationship between would be sustained?

**Author:** Thank you for excellent question. We included our hypothesis applied in dynamic tasks in the new version of manuscript.

**Reviewer:** I think the authors need to discuss how generalizable these findings are to actual clinical practice. I would argue that in an athletic or highly active population that movement biomechanics of the participant play into this moderation based on dynamics of the system during the task. I would suggest adding a paragraph to discuss the generalizability of the findings to practice.

**Author:** We have inserted it at the end of the Discussion section in the revised version.
